# Association between hyperlipidemia and nephrolithiasis: A comprehensive bioinformatics analysis deciphering the potential common denominator pathogenesis

**DOI:** 10.1371/journal.pone.0321734

**Published:** 2025-04-17

**Authors:** Zhikai Su, Zhenjie Ling, Haoqiang Chen, Lei Hu, Songtao Xiang, Qian Li, Jianfu Zhou

**Affiliations:** 1 Guangzhou University of Chinese Medicine, Guangzhou, China; 2 Department of Urology, The Second Affiliated Hospital of Guangzhou University of Chinese Medicine, Guangzhou, China; 3 Chinese Medicine Syndrome Research Team, The Second Affiliated Hospital of Guangzhou University of Chinese Medicine, Guangzhou, China; University of Helsinki: Helsingin Yliopisto, FINLAND

## Abstract

**Objective:**

Evidence suggests that nephrolithiasis and hyperlipidemia are linked. The study is designed to identify diagnostic biomarkers for nephrolithiasis in conjunction with hyperlipidemia using bioinformatics analysis, while exploring the potential common denominator pathogenesis.

**Methods:**

The NCBI Gene Expression Omnibus (GEO) database provided separate datasets for nephrolithiasis and hyperlipidemia. We employed the R limma package to detect differentially expressed genes (DEGs), which were subsequently analyzed for enrichment using Gene Set Enrichment Analysis (GSEA), Gene Ontology (GO), and Kyoto Encyclopedia of Genes and Genomes (KEGG) pathways. Immune cell infiltration was analyzed by the CIBERSORT method. The WGCNA-R package clustered genes with similar expression profiles, followed by an analysis of the associations between the modules and specific traits or phenotypes. The STRING database was utilized to establish a protein-protein interaction (PPI) network and key functional modules, which were then analyzed using Cytoscape software. Diagnostic genes for both diseases were screened from core hub genes using least absolute shrinkage and selection operator (Lasso) regression. Subsequently, we generated receiver operating characteristic (ROC) curves to validate the predictive ability of these diagnostic genes for diagnosing nephrolithiasis in combination with hyperlipidemia. Lastly, the Network Analyst platform facilitated the construction of transcription factor-gene (TF-gene) and TF-miRNA regulatory networks.

**Results:**

Based on datasets of nephrolithiasis and hyperlipidemia, we identified 167 DEGs and 74 hub genes through WGCNA. Using PPI networks and machine learning techniques, we recognized three frequently diagnostic genes (HSP90AB1, HSPA5, and STUB1), which demonstrated high diagnostic validity. The functional enrichment of these three diagnostic genes primarily involved pathways related to cellular metabolism.

**Conclusions:**

Our study identified three candidate diagnostic genes that can predict nephrolithiasis in conjunction with hyperlipidemia, providing a solid foundation for further exploration into the pathogenesis of nephrolithiasis and hyperlipidemia.

## Introduction

Urolithiasis is a highly prevalent condition and a leading cause of hospitalization in urology worldwide. A cross-sectional study using ultrasound revealed a prevalence rate of renal stones at 6.4%, with an age- and sex-adjusted rate of 5.8%. Specifically, the prevalence rate is 6.5% for males and 5.1% for females [1]. A meta-analysis of nephrolithiasis subgroups indicated that the highest prevalence rates in Guangdong (12.7%) and Guangxi (10.3%). Additionally, prevalence rates were higher in the developed eastern regions compared to the western regions, showing significant regional and provincial distinctions [2]. Despite a decline in the age-adjusted prevalence of urolithiasis among Chinese men and women over the past three decades, the crude prevalence in Chinese women has been increasing [3]. Worldwide prevalence of kidney stones is increasing. Despite successful therapeutic options such as percutaneous nephrolithotomy (PCNL), ureteroscopy and extracorporeal shock wave lithotripsy (ESWL) [4,5], the recurrence rate of renal calculus remains high, reaching up to 50% within 5–10 years after the first episode [6]. This imposes a significant burden on healthcare systems and socioeconomic factors [7]. Therefore, clarifying the key factors and molecular mechanisms underlying the formation and recurrence of nephrolithiasis holds great clinical significance and research value.

Hyperlipidemia, characterized by dysregulated lipid profiles, remains a major global health concern, contributing to metabolic syndrome and associated comorbidities [8]. Despite the significant decline in total cholesterol levels among populations of many developed countries [9], the overall prevalence of dyslipidemia in China remains relatively high, at 35.6% [10].A national cross-sectional study among adult males revealed an upward trend in total cholesterol and LDL cholesterol levels, along with low awareness, treatment, and control of hyperlipidemia [11]. Research has shown a positive association between unhealthy lifestyles, high-protein, high-fat diet intake, and the development of hyperlipidemia [12]. Additionally, hyperlipidemia acts as a key contributor to the risk of numerous diseases.

Recently, growing attention has been given to the link between lipid metabolism disorders and the prevalence of urolithiasis, suggesting a possible connection between circulating lipids and urolithiasis development [13,14]. A longitudinal study conducted in Taiwan found that individuals with hypertriglyceridemia (67–93 mg/dL) had a 1.463-fold increased risk of kidney stone disease, whereas low HDL-C could prevent kidney stone disease. Furthermore, Chol/HDL-C ratio exceeding 3.64 was linked to a 1.381-fold increased likelihood of developing kidney stones [15]. An animal experiment conducted by Chan Jung Liu [16] demonstrated that statins reduced the number of stones in rats with hydroxyproline water-induced formation of hyperoxic calcium oxalate renal calculi by ameliorating hyperlipidemia in rats. Moreover, statins facilitated the conversion of calcium oxalate to Calcium phosphate There is substantial evidence indicating a direct link between high serum triglyceride levels and an increased likelihood of developing urolithiasis [17,18]. However, there is a lack of relevant studies both domestically and abroad regarding the biological processes through which lipid metabolism disorders promote the development of nephrolithiasis. Therefore, exploring the intrinsic mechanisms linking lipid metabolism disorders and nephrolithiasis may present fresh perspectives on the prevention, therapy, and management of nephrolithiasis recurrence.

Given the current challenges in treating kidney stones and preventing their recurrence, along with the strong correlation between lipid metabolism disorders and kidney stone occurrence, this research seeks to explore the transcriptomics and proteomics associated with nephrolithiasis and hyperlipidemia. The objective is to identify diagnostic biomarkers related to the disease through a series of biosignature analyses, elucidate the biological processes underlying the disease, and construct the TF-gene regulatory network of pivotal genes as well as the TF-miRNA regulatory network of hub genes.

## Methods

### Data download

We downloaded gene expression data for nephrolithiasis and hyperlipidemia from the GEO database (https://www.ncbi.nlm.nih.gov/geo). The GSE73680 dataset, based on the GPL17077 platform, contains renal papillary tissue from 36 subjects who underwent endoscopic procedures (29 with the disease and 7 healthy controls). To assess diagnostic efficiency, we downloaded the GSE117518 dataset based on GPL21827, which includes renal papillary tissue from 6 patients (3 with the disease and 3 healthy controls). Randall’s plaque tissue was collected from patients undergoing percutaneous nephrolithotomy for renal calculus, while normal renal papillary tissue was obtained from patients with kidney neoplasms who underwent nephrectomy as well as from renal papillary tissue not invaded by the tumor. For hyperlipidemia, we utilized the GSE6054 dataset, based on the GPL570 platform, which consisted of 23 samples, of which 10 represented patients diagnosed with hyperlipidemia and 13 were healthy controls. The samples were obtained from blood, and gene expression profiles of isolated monocytes were detected using Affymetrix microarrays. The GSE13985 dataset (based on the GPL570 platform) including 5 hyperlipidemia samples and 5 healthy controls was additionally downloaded to assess diagnostic performance.

### Identification of DEGs

The raw expression matrix was standardized using R software (4.3.0). The “limma” R package [19] was employed to detect differentially expressed genes (DEGs) in the GSE73680 and GSE6054 datasets by comparing healthy control samples with disease groups. Statistically significant DEGs were identified based on the following criteria: p-value < 0.05, |log FC| ≥ 0.9. The R packages heatmap and ggplot2 were used to generate volcano plots of the Statistically significant DEGs and heat maps of the top 150 genes. The Bioladder Bioinformatics Online Analysis Platform (https://www.bioladder.cn/web/#/chart/17) was used to identify common DEGs between the two training datasets.

### Functional enrichment analysis

To achieve a more comprehensive understanding into the possible functions of the overlapping DEGs between the two training datasets, the Metascape database [20] (www.metascape.org) was utilized to conduct pathway analyses for Gene Ontology (GO) [21] and Kyoto Encyclopedia of Genomes (KEGG) [22]. The results of the enrichment analyses were uploaded to the Bioinformatics Cloud Platform (http://www.bioinformatics.com.cn) for visualization.

### Gene set enrichment analysis (GSEA)

GSEA [23] (version 4.3.3) was employed to conduct functional enrichment analysis for KEGG. The gene set parameters were configured as follows: gene names were chosen as the expression dataset, and ‘c2.cp.kegg_medicus.v2023.2.Hs.symbols.gmt’ was selected as the gene set database. The alignment value for calculating the Normalized Enrichment Score (NES) was set to the default of 1000. The maximum and minimum sizes for excluding larger and smaller gene sets were kept at the default values of 500 and 15, respectively. Signaling pathways with a p-value < 0.05 were considered enriched.

### Immune cell infiltration analysis using CIBERSORT

Based on the overlapping DEGs between the two training datasets, the immune cell composition in tissue samples was assessed using the CIBERSORT algorithm [24]. The analysis utilized the LM22 leukocyte signature matrix, which includes 22 immune cell subtypes. To ensure robust results, 1,000 permutations were performed. Only results with a CIBERSORT p-value below 0.05 were deemed significant and included in the analysis. A matrix of immune cell fractions was generated, and correlations between the 22 immune cell types were visualized using the R package “corrplot”. The “vioplot” package in R was used to visualize differences in immune cell infiltration between the experimental and control groups.

### A correlation analysis between overlapping DEGs and immune cells

To explore the immune mechanism involved in nephrolithiasis and hyperlipidemia development, we utilized the R software to run Spearman’s rank correlation analysis of the overlapping DEGs and immune cells. Besides the results were visualized with the “ggplot2” package.

### Weighted gene co-expression network analysis (WGCNA)

WGCNA [25] is a method in bioinformatics aimed at defining gene association patterns across multiple samples. It organizes genes with similar expression patterns and assesses the association between modules and distinct traits or phenotypes. The WGCNA-R package was employed to build co-expression networks for all genes in the dataset, and the algorithm identified the 10,000 genes exhibiting the highest variability for subsequent analysis. To estimate the network connectivity, the Weighted Neighborjoint matrix was converted to Topology Overlaymatrix (TOM), and hierarchical clustering was employed to construct its cluster tree structure. The branches of the clustering dendrogram correspond to distinct gene modules, with each color indicating a different module. Genes exhibiting comparable functions were clustered into a single module, with numerous genes being divided into various modules based on their weighted correlation coefficients. Many modules were closely related to nephrolithiasis or hyperlipidemia. Consequently, we proceeded with gene screening based on gene significance (GS) and module membership (MM). We used |MM| >0.8 and |GS| >0.5 as criteria to select key expressed genes in the hub module.

### Construction of protein-protein interaction (PPI) network and hub genes

The PPI network plays a vital role in functional biology research by mapping candidate genes (CGs) to publicly available PPI data to uncover pathways involving these genes [26]. Cytoscape [27], a tool available for the visualization, analysis, and construction of biological networks, was utilized to construct a PPI network of overlapping DEGs using the STRING database [28] (version 12.0, https://string-db.org). Co-expression networks were generated with Cytoscape (version 3.9.1), and Cytoscape’s MCODE plugin was utilized with its default parameters (Degree Cutoff: 2, Node Score Cutoff: 0.2, K-Core: 2, Max Depth: 100) to identify sub-networks of PPIs among the common CGs. Using the CytoHubba plugin algorithm [29], crucial genes within the PPI network were identified by applying six topology analysis methods (MCC, MNC, Degree, Closeness, Radiality, and EPC), with visualization achieved through upsetR. Bioladder Bioinformatics Online Analysis Platform (https://www.bioladder.cn/web/#/chart/17) was utilized to identify core hub-genes from the candidate genes derived through CytoHubba and MCODE. Four core hub genes were then input into GeneMANIA--an online tool) [30] (http://genemania.org) for analyzing gene co-expression networks, exploring interactions with core hub genes related to nephrolithiasis and hyperlipidemia, and predicting and visualizing gene set functions.

### Feature selection using the least absolute shrinkage and selection operator

Lasso is a widely-used regression technique that employs an ℓ1 penalty to obtain a sparse solution [31]. The “glmnet” package [32] in R was utilized to conduct least absolute shrinkage and selection operator (LASSO) regression, aiming to identify the most effective predictors for nephrolithiasis and hyperlipidemia.

### Prediction performance in validation cohorts

To validate the precision of these diagnostic genes, we retrieved GSE117518 for nephrolithiasis and GSE13985 for hyperlipidemia for external validation purposes. We obtained the raw data for both datasets from the GEO database and proceeded with normalization. For GSE117518, we used 2 healthy control samples and 3 nephrolithiasis patient samples. Validation was performed on 5 healthy controls and 5 patients with hyperlipidemia for GSE13985. Box-and-whisker plots and area under the ROC curve (AUC) were calculated for the diagnostic gene expression patterns of the validation cohort.

### Identification of relevant transcription factors and TF-miRNA regulatory network

To explore potential transcription factors (TFs) that might regulate diagnostic genes, the Network Analyst 3.0 online tool [33] from Homo sapiens (https://www.networkanalyst.ca) was employed to predict transcription factors (TFs) utilizing the ENCODE database, which holds microarray sequence data for numerous TFs. Cytoscape was used to complete the mapping of the TF-gene regulatory network.

### Ethics approval

This study did not involve human participants, interventions, or data collection directly from individuals. All data used in this study were obtained from publicly available online databases ‘GEO (Gene Expression Omnibus)’. GEO database provide fully anonymized datasets and ensure compliance with ethical standards, including obtaining necessary approvals and consents from original participants, as specified in their data usage policies. As the analysis involved secondary use of de-identified and publicly accessible data, ethical approval and informed consent were not required for this study.

## Results

### Information from GEO

**Fig 1:**
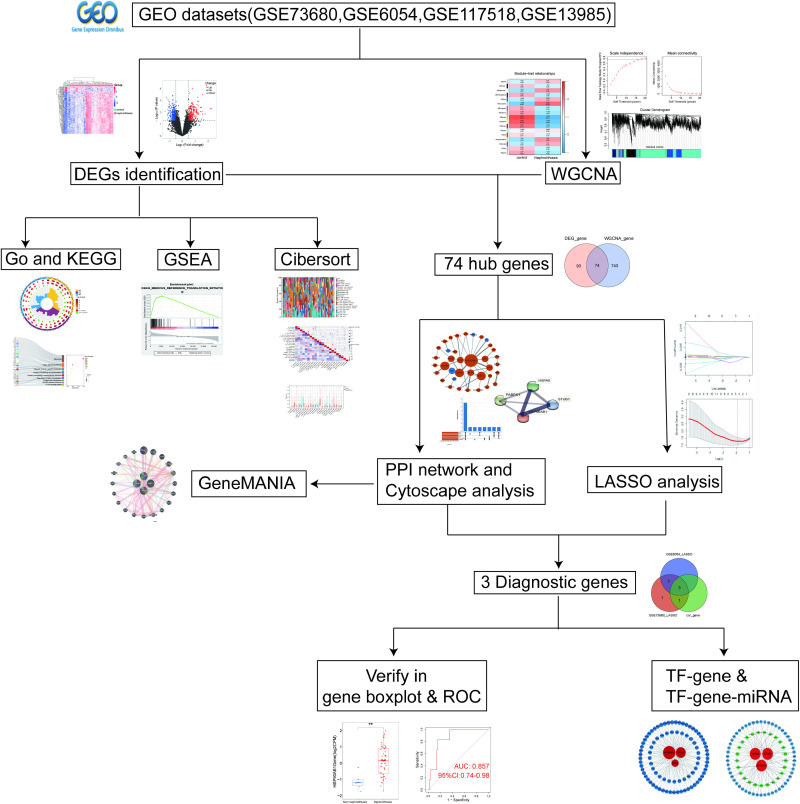
The complete research workﬂow.

Based on our inclusion criteria, four datasets were screened for exploratory analyses: GSE73680, GSE117518, GSE6054, and GSE13985. GSE73680 served as the discovery cohort for nephrolithiasis, while GSE6054 was designated as the discovery cohort for hyperlipidemia. Additionally, GSE117518 and GSE13985 were the validation cohorts for nephrolithiasis and hyperlipidemia respectively. The following is the complete research workflow (Fig 1).

### Identification of differentially expressed genes

**Fig 2:**
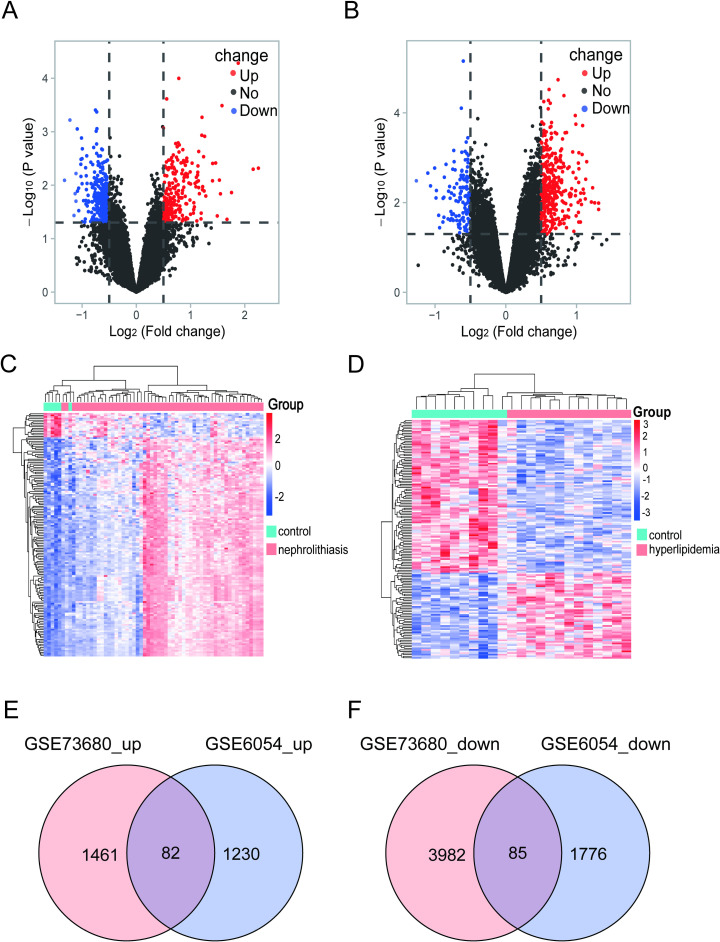
Volcano Map, Heatmap of DEGs, and Shared Gene Identification:(A) Volcano map: Significant gene expression changes in GSE73680. (B) Volcano map: DEGs in GSE6054, with red for up-regulated, blue for down-regulated, and black for unchanged genes. (C) Heatmap: Top 150 DEGs in GSE73680. (D) Heatmap: Top 150 DEGs in GSE6054. (E) Venn diagram: Overlap of up-regulated DEGs between GSE73680 and GSE6054. (F) Venn diagram: Overlap of down-regulated DEGs between the two dataset.

In the nephrolithiasis dataset GSE73680, we identified 5,610 DEGs. 1,543 DEGs were upregulated, while 4,067 DEGs were downregulated (Fig 2A). For GSE6054, 3,173 DEGs were identified, of which 1,312 were up-regulated and 1,861 were down-regulated (Fig 2B). The differential genes in these two groups were visualized using heatmaps (Fig 2C and 2D). Venn diagrams indicated that there were 82 overlapping co-upregulated genes (DEGs-up) and 85 co-downregulated genes (DEGs-down) between the nephrolithiasis and hyperlipidemia cohorts (Fig 2E and 2F).

### Functional enrichment analysis of gene set

**Fig 3:**
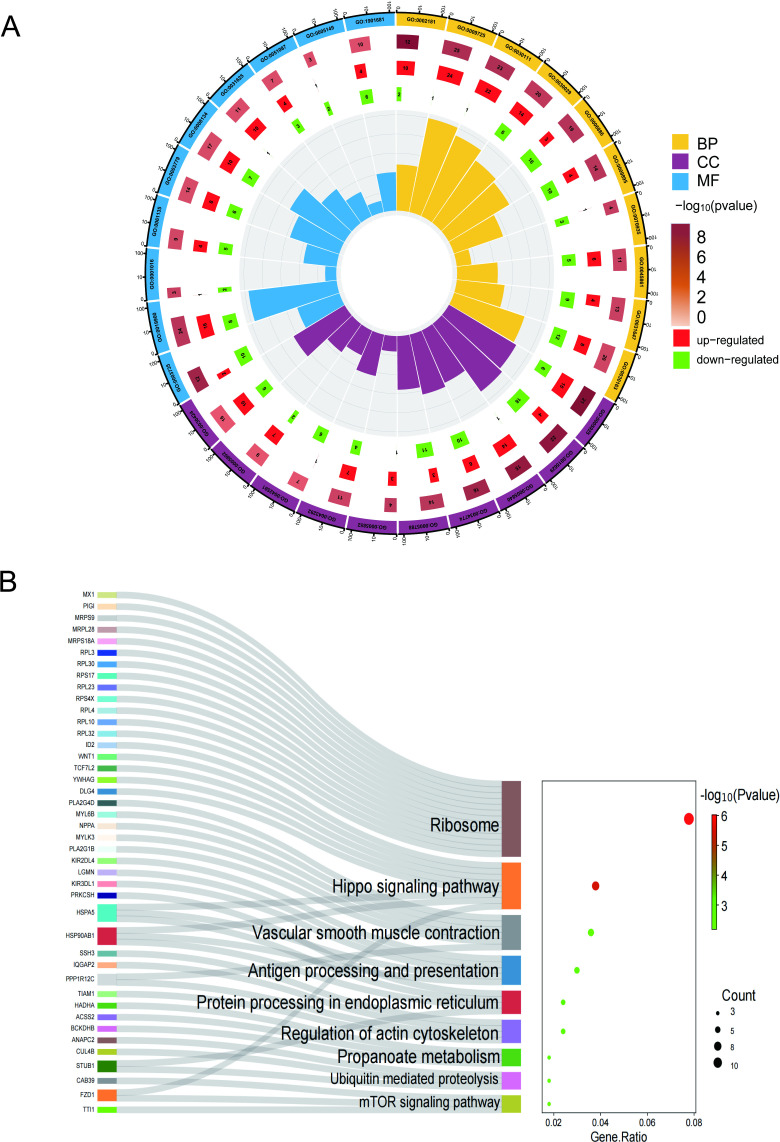
GO and KEGG analysis of shared genes. (A) GO enrichment analysis. (B) KEGG enrichment analysis.

Many of the enrichment pathways in Gene Ontology (GO) are associated with the regulation of the internal environment. The top 10 enriched GO pathways are shown in Fig 3A. Molecular Function (MF) analysis indicated that DEGs were primarily involved in ribosomal structural components (GO:0003735) and kinase binding (GO:0019900). For Cellular Component (CC) ontology, these genes are largely located in focal adhesion (GO:0005925) and the actin cytoskeleton (GO:0015629). As for the Biological Process (BP) category, genes were mainly enriched in cytoplasmic translation (GO:0002181), response to hormone (GO:0009725), regulation of Wnt signaling pathway (GO:0030111), actin filament-based process (GO:0030029), and intracellular protein transport (GO:0006886). KEGG enrichment analysis confirmed that the pathways of DEGs were enriched in the Hippo signaling pathway, protein processing in the endoplasmic reticulum, and ribosome signaling pathways (Fig 3B).

### Gene set enrichment analysis (GSEA)

**Fig 4:**
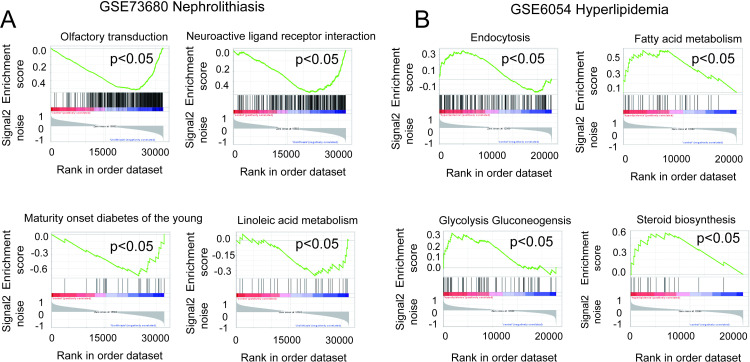
(A) GSEA plots showing the top four significantly enriched KEGG pathways in GSE73680 dataset (p-value < 0.05). (B) GSEA plots showing the top four significantly enriched KEGG pathways in GSE6054 dataset (p-value < 0.05). Screening criteria: Biological functions with a p-value < 0.05.

The metabolic patterns of nephrolithiasis and hyperlipidemia appear distinct yet interconnected. GSEA was conducted to evaluate the signaling pathways involved in DEGs. The results showed that pathways associated with kidney stones were significantly downregulated, including olfactory transduction, neuroactive ligand-receptor interactions, maturity-onset diabetes of the young, and linoleic acid metabolism (Fig 4A). Conversely, pathways associated with hyperlipidemia were significantly upregulated, including endocytosis, fatty acid metabolism, glycolytic gluconeogenesis, and steroid biosynthesis (Fig 4B). These results indicate that DEGs in the GSE73680 and GSE6054 are engaged in the regulation of metabolic functions in nephrolithiasis and hyperlipidemia.

### Immune cell infiltration

**Fig 5:**
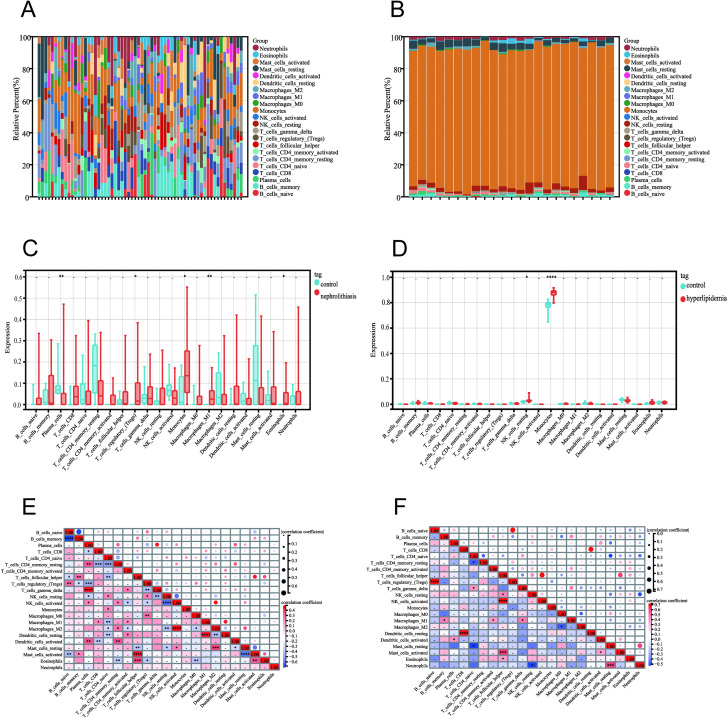
Distribution and visualization of immune cell infiltration. (A, B) Histogram showing the proportions of 22 immune cell subpopulations in the nephrolithiasis-GSE73680 cohort and the hyperlipidemia-GSE6054 cohort. (C, D) Immune cell infiltration patterns were visualized using boxplots for the nephrolithiasis-GSE73680 and hyperlipidemia-GSE6054 cohorts, with red indicating nephrolithiasis/hyperlipidemia patients and blue indicating healthy controls. (E, F) Immune cell fraction correlations were visualized in a matrix, with red for positive, white for neutral, and blue for negative correlations. The size of the circle also directly reflects the degree of correlation. * p-value < 0.05, ** p-value < 0.01, *** p-value < 0.001, **** p-value <0.0001, ns, no significance.

Differences in immune cell composition were examined between nephrolithiasis and normal control tissues. There were significant differences in immune cell composition between groups. Compared to the control group, the nephrolithiasis group exhibited a lower proportion of plasma cells (p < 0.01), while the proportions of regulatory T cells (p=0.02), monocytes (p=0.04), M1 macrophages (p<0.01), and eosinophils (p=0.02) were significantly higher (Fig 5A and 5C). In the hyperlipidemia group (Fig 5B and 5D), the proportions of resting NK cells (p=0.02) and monocytes (p<0.0001) were elevated, with monocytes showing the most pronounced increase. Nephrolithiasis and hyperlipidemia exhibited similar differential trends in comparison to normal samples, according to the differential expression analysis. Compared to healthy human samples, monocytes were notably increased in nephrolithiasis and hyperlipidemia. This indicates that nephrolithiasis and hyperlipidemia may involve inflammatory responses, immune dysregulation, and metabolic disorders.

The correlation analysis of 22 immune cell types used red for positive correlations, blue for negative correlations, and circle size to represent correlation strength. The results in Fig 5E indicate significant synergistic interactions in nephrolithiasis between T cells gamma delta and plasma cells, activated mast cells and T cells follicular helper, and M2 macrophages and activated NK cells. Conversely, the strongest competitive effect was observed between memory B cells and naive B cells. In hyperlipidemia (Fig 5F), regulatory T cells and naive B cells, resting dendritic cells and CD8 T cells, and activated NK cells and T cells follicular helper exhibited pronounced synergistic interactions. In contrast, the strongest competitive effect was observed between resting mast cells and naive CD4 T cells.

### Identification of co-expression gene modules

**Fig 6:**
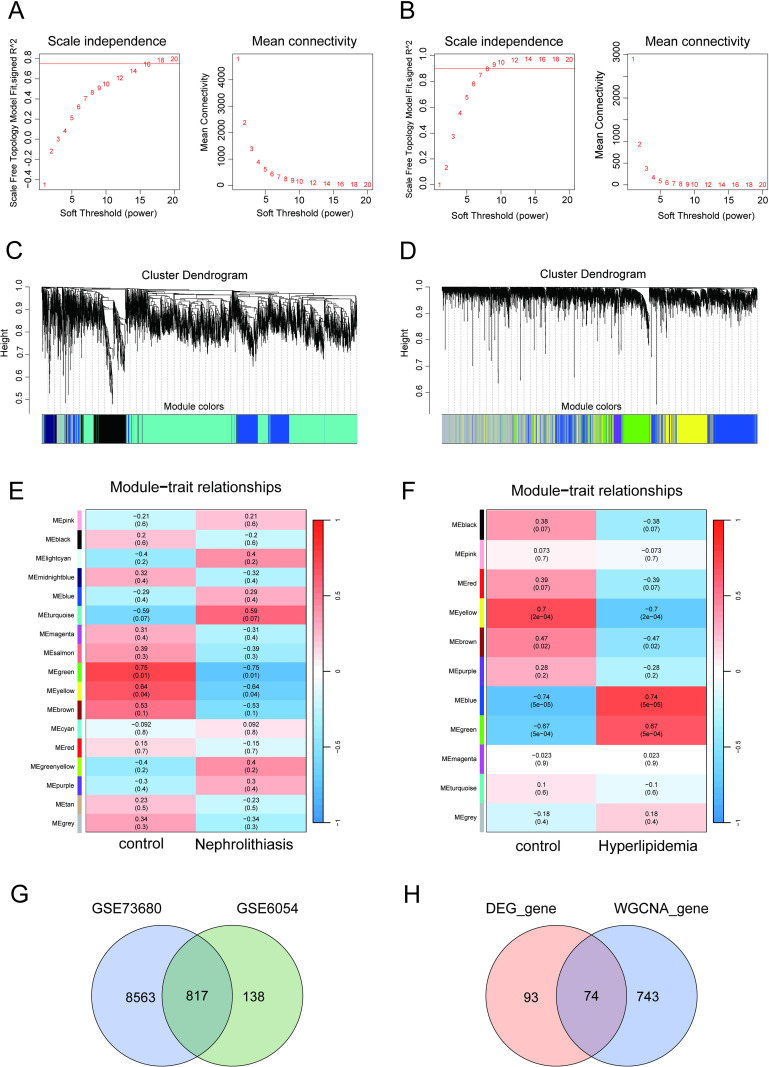
(A, B) Network topology: Assessment of soft thresholds (β) to achieve scale independence and mean connectivity. (C, D) Gene dendrograms: Module assignments based on dynamic tree cut clustering. (E, F) Module-trait relationships: Heatmap showing correlations between module eigengenes (MEs) and clinical traits with p-values. (G) Key module genes: Shared modules identified in both GSE73680 and GSE6054 datasets through WGCNA. (H) Common key module genes: Genes found in common DEGs and WGCNA.

To more thoroughly investigate the link between the disease and overlapping DEGs, we employed WGCNA with differential expression analysis across the two groups. We constructed a co-expression network by applying a soft-threshold method. Maintaining a scale-free topology within the co-expression network depends heavily on the parameter β. For the nephrolithiasis group, a fit index over 0.9 indicated a scale-free topology, and the β parameter was fixed at 16 (Fig 6A). The adjacency function was employed to generated an adjacency matrix, followed by the construction of hierarchical clustering using the TOM difference metric (Fig 6C). In total, 17 co-expression modules were recognized, with modules showing a P-value under 0.05 classified as essential. The turquoise module displayed a strong positive correlation, while the green, yellow, and brown modules exhibited significant negative correlations (Fig 6E). Within these four key modules in the nephrolithiasis group, 9,380 genes that matched |MM| > 0.8 and |GS| > 0.5 were further selected. Similarly, WGCNA was also conducted on the hyperlipidemia group, with β = 10 determined to be the optimal soft power value (Fig 6B). 19 modules were identified, with the blue and green modules demonstrating significant positive correlations, while the yellow module showed a strong negative correlation (Fig 6D and 6F). Using MM |>0.8 and | GS |>0.5 as standards, we further screened 955 genes from the genes of these three key modules in the hyperlipidemia group. These genes from the two sets of key modules may serve as candidate cell-specific markers. To investigate the common pathogenesis of nephrolithiasis and hyperlipidemia, we examined the intersection of the overlapping co-regulated genes and the genes identified through WGCNA. Fig 6G shows the key modular genes common to WGCNA for nephrolithiasis and hyperlipidemia, totaling 817. The common key modular genes in DEGs (GSE73680 and GSE6054) and WGCNA overlapped with 74 genes (Fig 6H).

### PPI and hub gene identification through MCODE and CytoHubba

**Fig 7:**
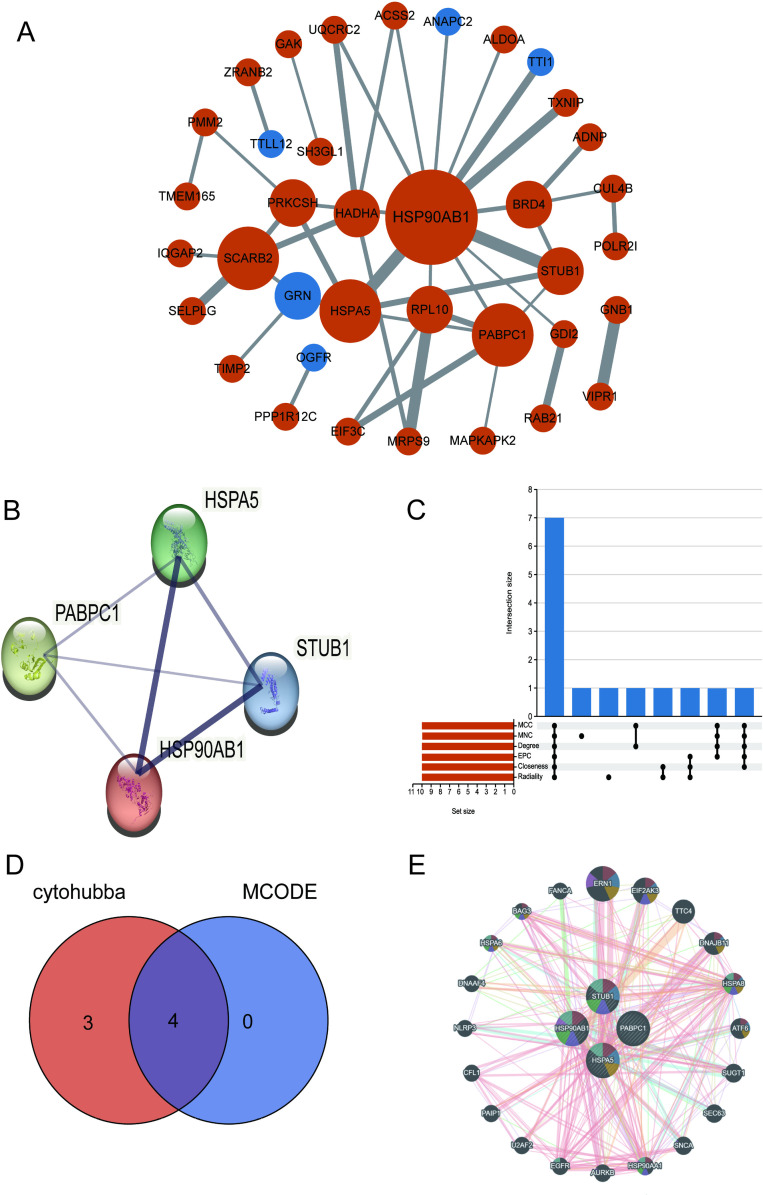
(A) The PPI network was mapped using the STRING visualization tool. (B) Identification of key gene module through MCODE plug-in in cytoscape. (C) Identifies hub genes using six Cytohubba algorithms (MCC, MNC, Degree, EPC, Closeness, Radiality). (D) Venn diagram highlighting four core hub genes common to CytoHubba and MCODE. (E) The GeneMANIA diagram displaying co-expression of core hub genes and their neighbors, with colors representing shared functions.

Seventy-four hub genes were imported into STRING, and unrelated genes were removed to derive a PPI network graph. This PPI comprised 37 nodes and 43 edges, with an interaction score above 0.4 (Fig 7A). The MCODE plugin in Cytoscape was used to identify hub gene modules, resulting in a core module containing 4 hub genes (Fig 7B). For topology analysis, we used the CytoHubba plugin within Cytoscape to pinpoint shared genes by applying six algorithms. The upsetR plot displayed seven intersecting genes from CytoHubba, namely HSP90AB1, PABPC1, HSPA5, STUB1, RPL10, PRKCSH, and BRD4(Fig 7C). By intersecting these 7 genes obtained from the CytoHubba plugin with the 4 genes obtained from the MCODE plugin, we identified 4 core hub genes, which were visualized using a Venn diagram (Fig 7D). GeneMANIA biofunctional analysis was used to explore genes with shared attributes and similar functions with the four core hub genes, illustrating the interactive functional association network among them. 20 molecules were found to be most closely related to the 4 overlapping DEGs, showing gene co-expression (8.01%), physical interaction (77.64%), predictive value (5.37%), co-localization (3.63%), genetic interaction (2.87%), and pathway involvement (1.88%) (Fig 7E). These genes are mainly functioned in peptidyl-serine modifications, cellular response to unfolded proteins, cellular response to heat, endoplasmic reticulum unfolded protein response, endoplasmic reticulum stress response, regulation of endoplasmic reticulum stress response, and protein folding.

### Identification of potential shared diagnostic genes via machine learning algorithms

**Fig 8:**
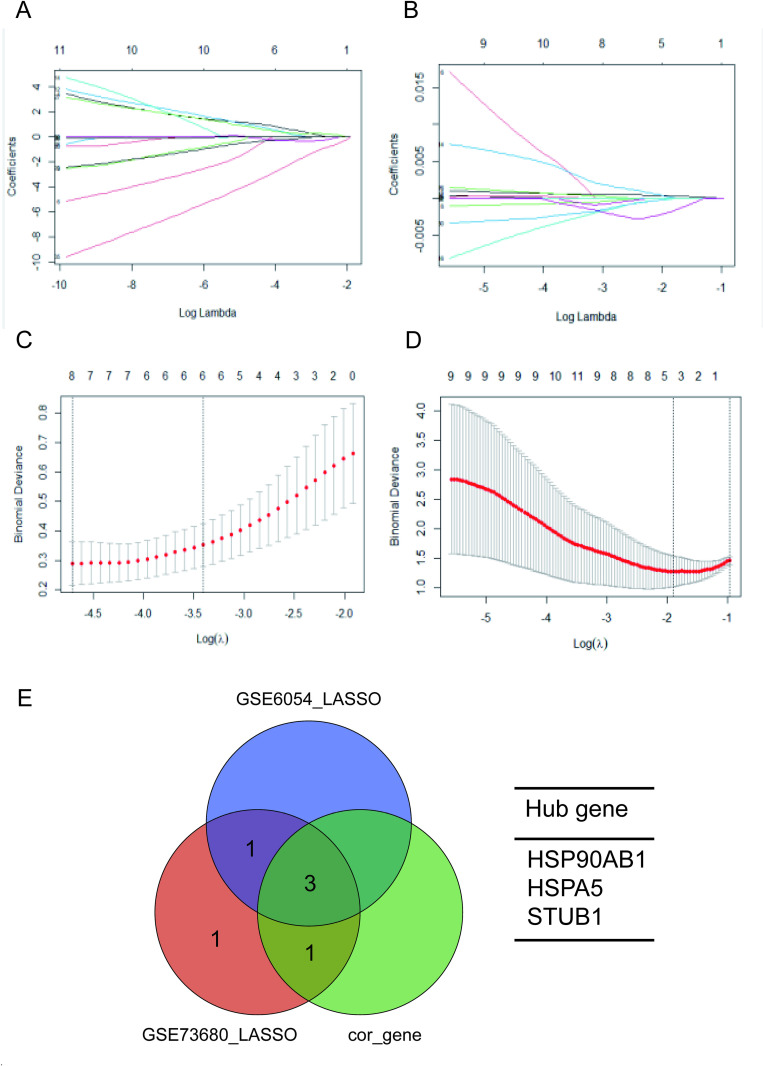
Core Gene Screening and Validation Using Machine Learning: (A) LASSO coefficient profiles for nephrolithiasis. (B) LASSO coefficient profiles for hyperlipidemia. (C, D) Ten-fold cross-validation was used to optimize (λ) in both nephrolithiasis and hyperlipidemia. (E) LASSO identified 6 core genes in nephrolithiasis, 4 in hyperlipidemia, and 3 diagnostic genes after intersection analysis.

Potential common diagnostic genes were identified using the LASSO regression algorithm. In GSE73680, 7 out of 37 core crossover genes were selected by LASSO with the optimal λ value set at 0.011 (Fig 8A and 8C). In the GSE6054 dataset, LASSO analysis pinpointed four out of the 37 core crossover genes with the optimal λ value of 0.011 (Fig 8B and 8D). Ultimately, the best common diagnostic biomarkers for nephrolithiasis and hyperlipidemia were identified as 3 diagnostic genes (HSP90AB1, HSPA5 and STUB1) (Fig 8E).

### Validation of diagnostic genes

**Fig 9:**
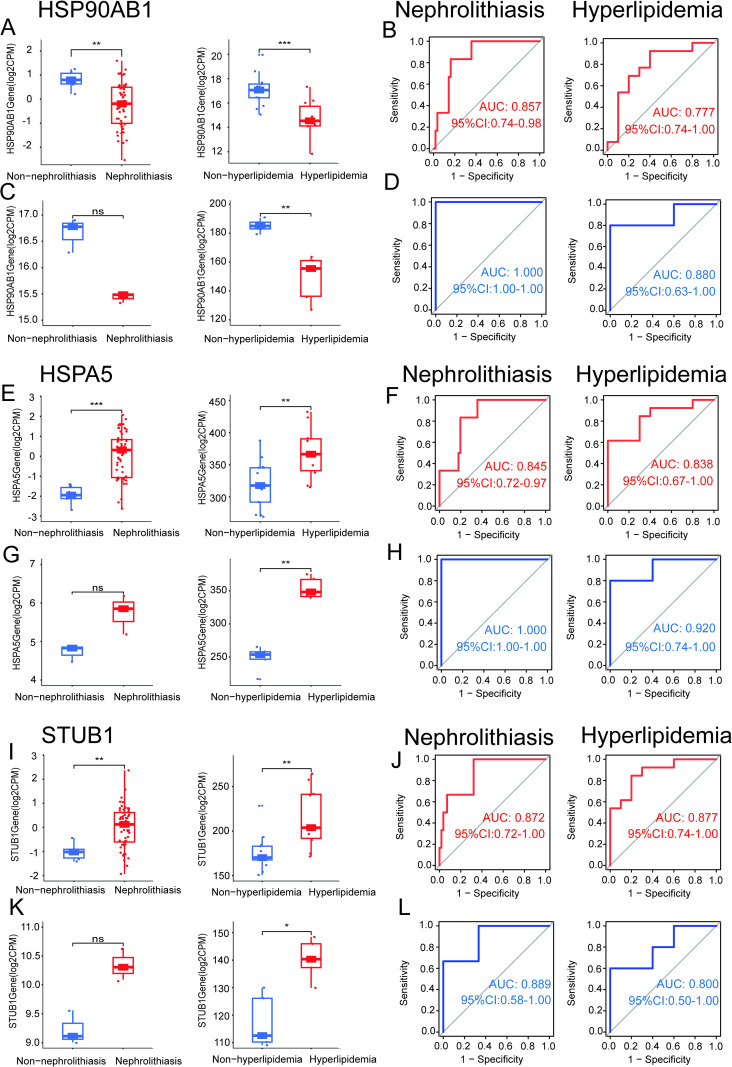
ROC Curve and Gene Boxplot for Diagnostic Testing of Diagnostic Genes. (A, C) HSP90AB1 gene boxplot for diagnosis and efficacy validation. (B, D) HSP90AB1 ROC curve for diagnostic performance. (E, G) HSPA5 gene boxplot for diagnosis and efficacy testing. (F, H) HSPA5 ROC curve for diagnostic accuracy. (I, K) STUB1 gene boxplot for diagnosis and efficacy analysis. (J, L) STUB1 ROC curve for diagnostic validation.

To further explore the diagnostic value of these biomarkers, we evaluated the predictive accuracy and discriminatory strength of the common diagnostic genes by analyzing the expression patterns of the three genes and their ROC curves. First, we examined the expression levels in nephrolithiasis and hyperlipidemia within the two discovery cohorts. The expression of HSP90AB1 was lower in both the nephrolithiasis group (P<0.01) and the hyperlipidemia group (P<0.0001) (Fig 9A). HSPA5 expression was higher in both the nephrolithiasis (P<0.001) and hyperlipidemia (P<0.01) groups compared to controls (Fig 9E). Similarly, STUB1 expression was elevated in the experimental group (P<0.01) (Fig 9I). Next, ROC analysis was performed to assess the specificity and sensitivity of the three diagnostic genes in diagnosing both diseases. For nephrolithiasis biomarkers, the three pivotal genes, HSP90AB1 (AUC=0.857), HSPA5 (AUC=0.845), and STUB1 (AUC=0.872), showed strong predictive performance. The same ROC analysis was performed for the hyperlipidemia group, with HSP90AB1 (AUC=0.777), HSPA5 (AUC=0.838), and STUB1 (AUC=0.877) also showing robust predictive performance (Fig 9B, 9F and 9J). In addition, we validated the reliability of HSP90AB1, HSPA5, and STUB1 as diagnostic genes for nephrolithiasis and hyperlipidemia through external validation. In both validation groups, the expression levels of these three diagnostic genes were consistent with those in the discovery cohort Fig 9C, 9G and 9K). HSP90AB1 showed low expressed in the validation set GSE117518 for nephrolithiasis, while the other two diagnostic genes were highly expressed, although the p values were not significant. Similar expression profiles were also observed in the hyperlipidemia validation set GSE13985. HSP90AB1 demonstrated good diagnostic accuracy in the nephrolithiasis validation cohort (AUC = 1.000) and in the hyperlipidemia validation cohort (AUC = 0.880) (Fig 9D). HSPA5 exhibited strong diagnostic potential in nephrolithiasis (AUC=1.000) and hyperlipidemia (AUC=0.920) (Fig 9H). Similarly, STUB1 correctly diagnosed nephrolithiasis (AUC=0.889) and hyperlipidemia (AUC=0.800) (Fig 9L). These findings confirm their potential as key diagnostic biomarkers for nephrolithiasis and hyperlipidemia.

### Construction of transcriptional regulation level regulatory networks

**Fig 10:**
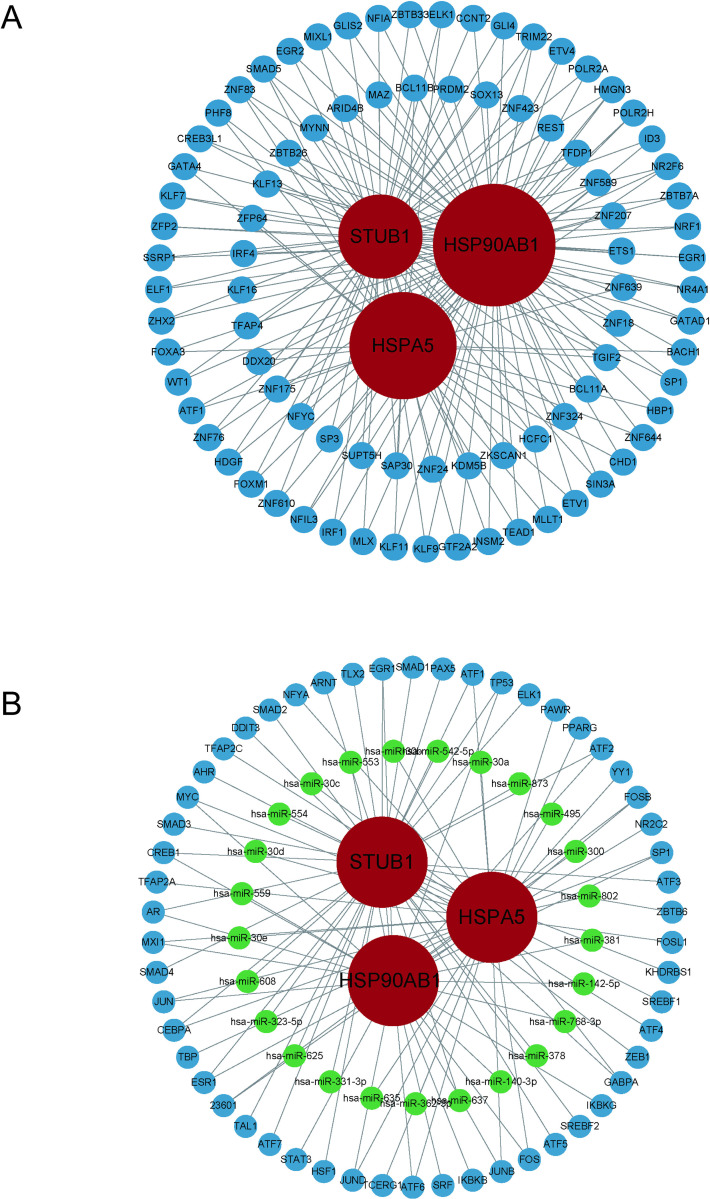
(A) TF-gene coregulatory network. (B) TF-gene-MiRNA co-regulatory network. Diagnostic genes are represented by red nodes, TFs by blue nodes, and miRNAs by green nodes.

It is helpful to reveal the biological processes underlying the pathogenesis of diseases through analyzing the interactions between transcription factors (TFs), miRNAs, and diagnostic genes. We constructed a TF-gene interaction network and a TF-miRNA co-regulatory network in our study, which were then imported them into Cytoscape for visualization and further analysis. The TF-gene network comprised 86 transcription factors, 3 diagnostic genes and 180 linkages (Fig 10A). The TF-miRNA co-regulatory network consisted of 98 connections involving 25 miRNAs, and 54 transcription factor genes interacting with the 3 diagnostic genes (Fig 10B).

## Discussion

A Polish study on obese children aged 3–18 years indicates that those with hypercholesterolemia and hypertriglyceridemia exhibit reduced urinary citrate excretion, elevated ionic calcium levels, and a greater propensity for developing kidney stones [34]. Additionally, multiple clinical studies have found a positive association between hyperlipidemia and nephrolithiasis [35–37] These findings suggest that hyperlipidemia significantly impacts the occurrence of nephrolithiasis. Another study confirmed that patients without a history of urolithiasis who received statin therapy had lower lipid parameters (LDL, TG, cholesterol) and a significantly reduced risk of developing new urolithiasis compared to those who did not receive statin therapy [38]. The potential mechanism may involve statins reducing autophagy-ERS responses, renal injury, and crystal deposition levels, thereby decreasing the formation of calcium oxalate kidney stone formation and protecting the kidneys [39]. Meanwhile, Kaisaier Aji et al. identified KLK1 and MMP10 as key genes associated with kidney stone formation using WGCNA and machine learning. While these genes have been implicated in metabolic pathways related to kidney stones, their potential connection to hyperlipidemia remains unexplored [40].

Despite these findings, as far as we know, transcriptomic data have not yet been utilized to evaluate potential diagnostic biomarkers among healthy controls, nephrolithiasis, and hyperlipidemia. Therefore, to explore the connection between nephrolithiasis and hyperlipidemia, we conducted bioinformatics and enrichment analyses by merging independent datasets for both diseases, leading to the identification of three diagnostic genes (HSP90AB1, HSPA5, and STUB1), offering new insights and potential directions for future research. The following sections will elaborate on the relationships between these three genes, nephrolithiasis and hyperlipidemia.

HSP90AB1, commonly known as HSP90β, is a heat shock protein family member with molecular chaperone activity. Chaperone proteins aid in correct protein folding and maintain protein stability by binding to client proteins, especially during cellular stress [41]. Our study suggests that HSP90AB1 is a potential diagnostic target for patients with hyperlipidemia and kidney stones, a conclusion that is partially supported by previous research. Nilubon Singhto et al. found that lower levels of HSP90β were positively correlated with kidney stone occurrence, indicating a possible association between HSP90AB1 and kidney stones. Additionally, HSP90 has been shown to promote nitric oxide production by endothelial nitric oxide synthase (eNOS), regulating renal vascular tension, sodium excretion, and urine concentration, which may affect the excretion of stone forming substances in urine and then indirectly participating in the formation of kidney stones [42]. Furthermore, Victoria Ramírez et al. applied the HSP90 inhibitor Radicicol to male Wistar rats and observed a decrease in renal blood flow and glomerular filtration rate, creating an environment conducive to calcium oxalate stone formation in the renal pelvis [43], further supporting our findings. Based on the research results, we speculate that the abnormal expression or function of HSP90β may lead to the dysfunction of renal tubular epithelial cells and increase the risk of crystal deposition. Similarly, Hsp90β may affect the survival and repair of renal tubular epithelial cells by regulating the expression of apoptosis related proteins. Increased apoptosis may lead to renal tubular injury and promote crystal nucleation and growth.

HSP90AB1 is also associated with hyperlipidemia. A reduction in HSP90AB1 levels can have a preventive effect on hyperlipidemia by maintaining the stability and activity of sterol regulatory element-binding proteins (SREBP), which play a crucial role in de novo lipogenesis (DNL) [44,45]. Based on the results of immune infiltration, we speculate that HSP90AB1 may contribute to hyperlipidemia by modulating monocyte activation and inflammatory responses. In hyperlipidemia, monocytes activated by oxidized low-density lipoprotein (oxLDL) differentiate into pro-inflammatory macrophages and release inflammatory cytokines. Additionally, it has also been shown that the knockout of HSP90AB1 in mice facilitates the degradation of mature sterol regulatory element-binding proteins (mSREBPs) via the Akt-GSK3β-FBW7 pathway. This significantly reduces neutral lipids and cholesterol levels, thereby decreasing de novo lipogenesis in hepatocytes [45].

Heat shock protein A5 (HSPA5), also known as glucose-regulated protein 78 (GRP78) is primarily responsible for refolding or degrading misfolded proteins in the endoplasmic reticulum to maintain low levels of unfolded proteins [46–48]. Although the direct link between HSPA5 and kidney stones and hyperlipidemia are not yet fully confirmed, several researches propose that HSPA5 may play a role in the pathogenesis of these diseases. Crystalline binding sites provide the foundation for crystallization, facilitating crystal deposition and kidney stone formation possible [49].Calcium serves as an essential component in the development of calcium oxalate stones, and endoplasmic reticulum stress-induced upregulation of GRP78 may lead to increased urinary calcium ion concentration, promoting kidney stone formation. The primary binding site for calcium ions is in the endoplasmic reticulum, and elevated cytosolic Ca^2+^ can disrupt the function of endoplasmic reticulum chaperones, inducing endoplasmic reticulum stress, activating the unfolded protein response (UPR), and subsequently upregulating GRP78 [50]. Several studies have shown that increased oxalate can stimulate the expression of GRP78, a marker of endoplasmic reticulum stress, leading to increased crystal deposition in the kidneys and creating a favorable environment for kidney stone formation [39,51,52]. Additionally, GRP78 may be involved in the oxidative stress process, creating conditions conducive to kidney stone formation. Rishi Bhardwaj et al. found that increased expression of the GRP78 marker indicates endoplasmic reticulum stress under hyperoxic conditions, accompanied by the appearance of calcium oxalate crystals [53].

Some attention has also been given to the relationship between HSPA5 and hyperlipidemia. In a cross-sectional analysis, serum GRP78/BiP levels were positively correlated with LDL cholesterol, non-LDL cholesterol, and triglycerides, suggesting that GRP78 may be associated with the occurrence of hyperlipidemia [54]. Additionally, reticulon 3 (RTN3) regulates triglyceride biosynthesis and storage through its interaction with heat shock protein family A (HSP70) member 5, which may be a mechanism for hyperlipidemia occurrence [55]. In conclusion, HSPA5 may play a shared role in the pathogenesis of nephrolithiasis and hyperlipidemia by regulating endoplasmic reticulum stress, oxidative stress, and inflammatory responses. In nephrolithiasis, HSPA5 may mediate renal tubular epithelial cell injury through endoplasmic reticulum stress, promoting calcium oxalate crystal deposition, while also exacerbating stone formation by modulating oxidative stress and the release of inflammatory cytokines. In hyperlipidemia, HSPA5 would contribute to lipid accumulation and metabolic dysregulation by influencing lipid metabolism-related signaling pathways including NF-κB and AMPK, as well as inflammatory responses. Both diseases involve the crosstalk of HSPA5-mediated endoplasmic reticulum stress-inflammatory axis and oxidative stress, suggesting that HSPA5 serves as a key molecular link between nephrolithiasis and hyperlipidemia.

STUB1 is a newly identified co-chaperone with ubiquitin ligase functio, involved in facilitating the degradation of misfolded proteins within cells. It encodes CHIP (C-terminal of Hsc70 Interacting Protein), an essential E3 ubiquitin-protein ligase that contributes to protein quality control and cellular homeostasis [56]. Studies have shown that overexpression of cystic fibrosis transmembrane conductance regulator (CFTR) can alleviate renal tissue damage and calcium oxalate deposition in mice, while STUB1 exacerbates calcium oxalate-induced renal damage by regulating CFTR ubiquitination and reactive oxygen species-mediated autophagy [57]. Calcium oxalate deposition and kidney injury would create favorable conditions for kidney stone formation. Additionally, STUB1 can indirectly influence kidney stone formation by influencing affecting aquaporin 2 (AQP2), which regulates urine volume and osmolality. The expression and regulation of AQP2 are primarily controlled by vasopressin-dependent proteins, whose half-life and abundance are influenced by AQP2 ubiquitination [58]. Animal experiments have found that CHIP gene knockout mice or CRISPR/Cas9-modified mice lacking CHIP E3 ligase function experience increased AQP2 levels, which affect kidney water regulation, decrease water intake and urine volume, and elevate urine osmolality [59]. Moreover, STUB1 cooperates with arginine vasopressin (AVP) to induce water reabsorption through principal cells of the collecting duct [60]. Based on existing reports, we hypothesize that STUB1 may promote kidney stone formation by increasing tubular reabsorption, urinary osmolality, and the accumulation of oxalate and calcium ions in the kidney. Although the relationship between STUB1 and hyperlipidemia has not been confirmed, research has indicated that upregulation of STUB1 may alleviate lipid droplet accumulation and steatosis in the liver of mice, improving non-alcoholic fatty liver disease [61]. Jie Luo et al found that upregulation of SREBP1 expression in chronic kidney disease models drives increased cholesterol synthesis [62]. Accordingly, we speculate that STUB1 may inhibit this process through ubiquitin-mediated degradation of SREBP1. If STUB1 function is impaired, it may not be able to effectively degrade SREBP, resulting in hyperactivation of the cholesterol synthesis pathway, thereby promoting lipid accumulation.

It is necessary to acknowledge the inherent limitations in our study. First, the currently available datasets related to nephrolithiasis and hyperlipidemia are indeed limited in size. Although these datasets represent the publicly available data in this field and have been used in prior literature, the limitations of the datasets have had a certain impact on the results of the research. Specially in the validation of diagnostic genes, the result may reflect dataset-specific characteristics or overfitting. Therefore, it is necessary for future studies to utilize larger datasets to enhance the statistical power and generalizability of our findings. Second, this experiment has not been validated at the cellular and animal levels, and prospective studies are required to confirm our results.

## Conclusion

In conclusion, this study identifies new diagnostic biomarkers for nephrolithiasis and hyperlipidemia using bioinformatics approaches and elucidates the potential common denominator pathogenesis. The identified biomarkers not only offer potential diagnostic tools but also provide a foundation for future therapeutic strategies targeting shared metabolic pathways in nephrolithiasis and hyperlipidemia. Prospective studies, including clinical trials and animal models, are warranted to validate these findings.
